# Spatiotemporal differentiation and driving mechanisms of high-level Grade A tourist attractions in the Yangtze River Delta City Cluster

**DOI:** 10.1371/journal.pone.0300181

**Published:** 2024-05-22

**Authors:** Mengchan Chen, Fangqin Yang, Jianwei Sun, Lingling Deng, Jing Luo

**Affiliations:** 1 School of Geographical and Environmental Sciences, Guizhou Normal University, Guiyang, China; 2 School of Economics, Guizhou University, Guiyang, China; 3 Key Laboratory for Geographical Process Analysis & Simulation of Hubei Province, Central China Normal University, Wuhan, China; Huazhong University of Science and Technology, CHINA

## Abstract

Herein, the spatial evolution characteristics of high-level Grade A tourist attractions in the Yangtze River Delta (YRD) urban agglomeration, from 2001 to 2021, are studied by comprehensively applying the nearest neighbor index, kernel density analysis, standard deviation ellipse, and spatial autocorrelation. High-level Grade A tourist attractions are investigated using the random forest model as the driving mechanism of the spatial pattern. Results show that 1) the spatial distribution of high-level Class A tourist attractions in the YRD city cluster has grown to be an agglomeration, and the high-density areas have evolved from “point-like dispersion to regiment-like combination,” gradually forming a B-shaped core density structure. 2) The spatial distribution comprises an overall “northwest–southeast” direction, a small counterclockwise rotation, the distribution of the center of gravity to the southwest migration, and the center of gravity from the territory of Suzhou City to the territory of Huzhou City. 3) The high-level Class A tourist attractions in the YRD city cluster as a whole show a strong positive spatial correlation, and the significantly clustered areas include three types: high-high (H-H), low-low (L-L), and low-high (L-H). 4) The spatial distribution of high, A-level tourist attractions in the YRD city cluster results from the combined action of the natural environment, resource endowment, socioeconomy, and policy background. Each element has a nonlinear and complex influence on the distribution of scenic spots.

## 1 Introduction

The “14^th^ Five-Year Plan” for Culture and Tourism Development in China implies that efforts will be made to improve the modern tourism system and achieve high-quality tourism development. As a strategic industry of the national economy, tourism plays a crucial role in stimulating domestic demand, solving employment problems, promoting the economy, and improving the environment [1,2]. The tourism industry of China is in a critical period as the national economy shifts gears, deepening the integration of culture and tourism, implementing national strategies, and adjusting the global tourism pattern. The growth scale of the traditional tourism industry is unable to cope with this complex change; alarming an urgent need to switch the focus, optimize the industrial structure, and improve the industry quality [3]. Class A tourist attractions are the primary material conditions for developing the tourism industry and core tourism products [4]. Its spatial distribution profoundly impacts the market competition and innovation of the industry [5], especially high-level tourist attractions, playing a leading role in regional tourism development. Being one of the most economically developed regions of China, the Yangtze River Delta (YRD) urban agglomeration is rich in tourism resources, having a huge tourism industry. In 2021, the total number of domestic tourists in the YRD urban agglomeration was >1.5 billion, and the total income from domestic tourism was >2 trillion yuan, accounting for >45% and >70% of the national ratio, respectively, making it the leading area of tourism development in China. Therefore, in the new era of high-quality tourism development, studying the spatial and temporal differences of high-level Grade A tourist attractions in the YRD city cluster and exploring the driving mechanism to optimize the spatial layout of tourist attractions, promote rational tourism development resources, facilitate the supply-side reform of tourism, and achieve high-quality tourism development is important.

International scholars’ research on tourist attractions began in the 1960s [6], primarily focusing on the analysis and classification [7,8], evaluation and competition [9–11], marketing and planning [12,13], tourists’ perceptions [14–16], and spatial distribution of scenic spots [17–24]. The spatial distribution of tourist attractions has become a new research hotspot in recent years. In the spatial distribution of scenic spots, Wilson constructed a large entropy-gravity model to measure it [25]; Judd confirmed the “line” distribution of urban tourist attractions [26]; Weaver applied the core-edge theory to the empirical study of scenic spots [27]; Ihab explored the influence on the spatial structure of tourism concerning cost and economic development [28]. Domestic scholars began to research tourist attractions in the 1980s, mainly focusing on scenic spot planning and development [29–31], operation and management [32,33], spatial structure [34–36], and tourists’ satisfaction [37,38]. In 2001, China formally implemented the quality assessment system for A-level tourist attractions; the scenic spot rating affects tourists’ judgment of the quality of scenic spots and is a key factor for tourists to assess the quality of tourist destinations. Therefore, Chinese scholars have researched Class A tourist attractions, focusing mainly on spatial distribution and evolution [39], as well as the influencing factors and mechanisms of the scenic spots [40,41]. Research methods have involved mathematical modeling [4], GIS spatial analysis [42], and geodetectors [43]. Multiple spatial scales have been employed, such as the nationwide [44], watersheds [5], regions [45], urban agglomerations [46], and provinces [47]. Research theories utilized the tourism land life cycle, point-axis, and core-edge theories.

Domestic and foreign scholars have achieved fruitful results in exploring the spatial pattern of tourist attractions, providing a solid theoretical reference and methodological support for this study. However, there are still several deficiencies: (1) Existing studies mainly analyze the spatial distribution characteristics of the scenic areas at a certain time cross-section from a static viewpoint, and a few studies have explored the spatial and temporal evolution of the scenic areas before 2016; however, after 2016, the number of Class A tourist attractions rose sharply, with the number of high-level Class A tourist attractions in the YRD city cluster in 2021 increasing by 74%, compared with 2016. (2) The random forest model, a machine learning algorithm effectively assessing the importance of variables, has been widely used to detect the importance of influencing factors in many fields, such as the spatiotemporal evolution of the structure of construction land and the spatiotemporal differentiation of the resilience of star-rated hotels, but it is seldom used to measure the influencing factors of the spatiotemporal differentiation of the scenic spots. (3) Though the YRD city cluster leads the Chinese tourism industry, there is a relative lack of tourism-related research, especially on the spatiotemporal differentiation and driving mechanisms of high-level Class A tourist attractions. Thus, this study takes 3A-level and above tourist attractions as high, A-level tourist attractions in the YRD city cluster as the research object, uses the GIS spatial analysis method and random forest model to analyze the law of spatial and temporal differentiation and the factors affecting the spatial and temporal evolution of these tourist attractions. The aim is to optimize the spatial structure of high-level Class A tourist attractions in the YRD city cluster, promote synergistic tourism development, and provide case studies to realize high-quality development of the tourism industry in the city clusters of China.

## 2 Research area, methodology, and data

### 2.1 Research area

**Fig 1:**
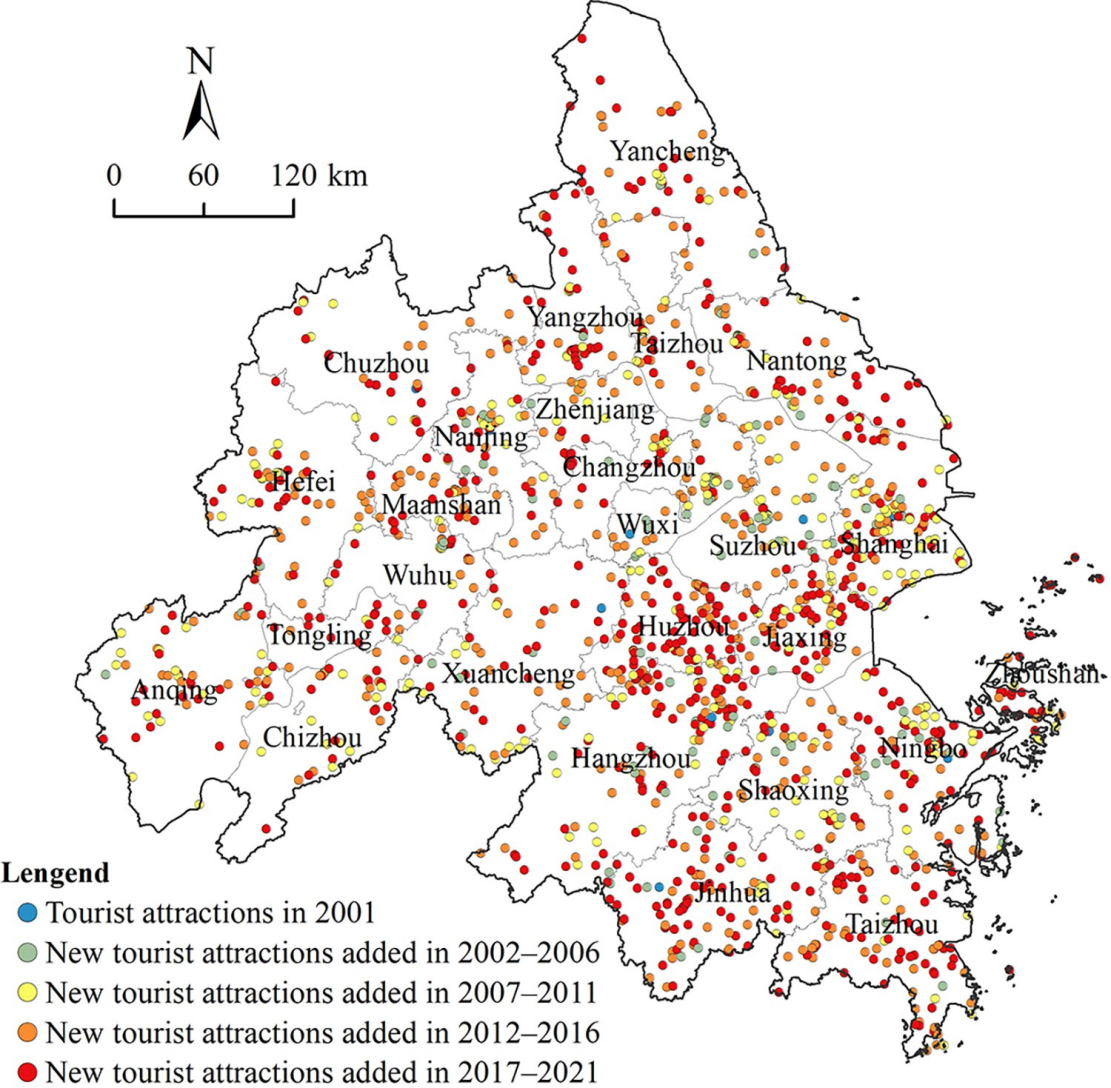
Spatial distribution of high-level Grade A tourist attractions in the YRD city cluster.

The YRD city cluster is located in the lower reaches of the Yangtze River in China, spanning 115° 45′–123° 25′ E and 28° 01′–34° 28′ N. According to the YRD City Cluster Development Plan approved by the State Council of China in 2016, the cluster includes 26 cities in the provinces and the cities of Suzhou, Zheijiang, Anhui, and Shanghai at the prefecture-level and above, with a land area of 211,700 km^2^. It is one of the urban agglomerations with the most complete industrial system and the most developed tourism industry of China. The YRD city cluster has a long cultural history. Jinling, Wuyue, and Hui cultures are intertwined, coupled with a well-developed water system and a temperate climate, which have nurtured many historical humanistic, and natural landscapes. The region is rich in tourism resources and endowed with excellent tourism. As of December 2021, there were 1,483 3A and above tourist attractions in the YRD city cluster, including 898 3A-level scenic spots, 540 4A-level scenic spots, and 45 5A-level scenic spots (Fig 1).

### 2.2 Research methodology

#### 2.2.1 Nearest neighbor index

The nearest neighbor index is a method for measuring the geospatial distribution pattern of “point elements” by determining their geospatial distribution pattern through the degree of mutual proximity between the objects under study [48], with the following formula:

$$R=\left(\sum_{i=1}^{n}\min d_{\mathrm{ij}}/n\right)/0.5\sqrt{A/n}$$ (1)

where *R* denotes the closest neighbor index; min*d*_*ij*_ is the Euclidean distance between any tourist attraction in the region and its closest neighboring tourist attraction; *n* is the total number of tourist attractions; and *A* is the geographical area of the YRD city cluster. *R* > 1 denotes a dispersed distribution of tourist attractions, *R* < 1 denotes a clustered distribution, and *R* = 1 denotes a random distribution.

#### 2.2.2 Kernel density estimation

KDE estimates the density of point or line elements with the help of moving cells, which effectively characterizes the agglomeration density dynamics of spatial unit elements [49], with the following formula:

$$f(x,y)=\frac{1}{nh}\sum_{i=1}^{n}k(\frac{d_{i}}{h})$$ (2)

where: *f*(*x*,*y*) is the density estimate of the tourist attraction at spatial location (*x*,*y*), $k(\frac{d_{i}}{h})$ is the kernel function, *d*_*i*_ is the distance of the tourist attraction at location (*x*,*y*) from the *i*th observation point, *n* is the number of sample points, and *h* is the bandwidth or smoothing parameter.

#### 2.2.3 Center of gravity model and standard deviational ellipse

The center of gravity model can be used to summarize the spatial center of gravity change trajectory of geographic elements [50], with the help of which this paper analyzes the center of gravity migration direction and distance of high-level Grade A tourist attractions in the YRD urban agglomerations with the following formulas:

$$X=\frac{\sum X_{i}W_{i}}{\sum W_{i}},\;Y=\frac{\sum Y_{i}W_{i}}{\sum W_{i}}$$ (3)

where *X* and *Y* denote the coordinates of the center of gravity of the tourist attractions, *X*_*i*_ and *Y*_*i*_ are the coordinates of each study unit, and *W*_*i*_ is the number of tourist attractions in the *i*th study unit.

The standard deviation ellipse expresses the distribution range and directional trend of geographic elements in the study area [51]. This paper conducts a preliminary investigation on the directionality, agglomeration, and spatial morphology of the distribution of scenic spots using this method.

#### 2.2.4 Spatial autocorrelation analysis

Spatial autocorrelation reflects the spatial correlation between the attribute values of geographic elements in a region and neighboring regions, including global spatial autocorrelation and local spatial autocorrelation [52]. Global spatial autocorrelation depicts the geographic dependence of the research object as a whole, which Moran’s I usually expresses with the following formula:

$$\mathrm{Moran}’s\;I=n\frac{\sum_{i=1}^{n}\sum_{j=1}^{n}w_{\mathrm{ij}}(x_{i}-\bar{x})(x_{j}-\bar{x})}{\sum_{i=1}^{n}\sum_{j=1}^{n}w_{\mathrm{ij}}\sum_{i=1}^{n}{\left(x_{j}-\bar{x}\right)}^{2}}$$ (4)

where *x*_*i*_ and *x*_*j*_ denote the number of tourist attractions in grids *i* and *j*, respectively, $\bar{x}$ is the average value, *w*_*ij*_ is the spatial adjacency weight matrix value of *i* and *j* within the grid, and *n* is the number of grids.

In contrast, local spatial autocorrelation visually identifies the degree of geospatial heterogeneity of the study object in a localized region [53], and the local Moran’s I is calculated as follows:

$$I_{i}=\frac{X_{i}‐\bar{X}}{S^{2}}\sum_{j=1}^{n}W_{ij}(X_{j}‐\bar{X})$$ (5)

where $S^{2}=\frac{1}{n}{\sum_{i=1}^{n}\left(X_{i}‐\bar{X}\right)}^{2}$. The meaning of the rest of the variables is the same as in the previous section. The local spatial autocorrelation can be categorized into four clustering patterns: high-high clustering (HH), low-low clustering (LL), high-low clustering (HL), and low-high clustering (LH).

### 2.2.5 Random forest

Random forest is a classification, tree-based machine learning algorithm proposed by Breiman in 2001 [54], which draws multiple samples from the original sample for decision tree modeling through the bootstrap resampling method and ultimately votes on the results after combining the predictions of multiple decision trees [54,55]. The algorithm is widely applied in classification and regression problems. The expression of this model for evaluating the importance of variables is

$$imp_{i}=\frac{1}{\mathrm{ntree}}\sum_{v=s_{xi}}\mathrm{Gain}(X_{i},v)$$ (6)

where *imp*_*i*_ denotes the contribution of the variable *X*_*i*_ to the regression model, expressed as *%IncMSE*, and the larger the value, the more important the variable. *S*_*xi*_ is the set of nodes that are split by *X*_*i*_ in the regression model of the *ntree* tree decision tree, and *gain* (*X*_*i*_,*v*) is the Gini information gain of *X*_*i*_ at the split node *v*.

### 2.3 Data sources

Data on high-level Class A tourist attractions in the YRD city cluster were obtained from the Ministry of Culture and Tourism of the People’s Republic of China (https://www.mct.gov.cn/) and the official websites of the Culture and Tourism Departments of 26 cities in the YRD region. The coordinate locations of the scenic spots were obtained through the Baidu coordinate picking system (https://api.map.baidu.com/) and GeoSharp was used for error correction. The National Platform for Earth System Science Data Sharing (http://www.geodata.cn) obtained the administrative district base map data. The temperature, river network, and DEM data were obtained from the Center for Resource and Environmental Science and Data of the Chinese Academy of Sciences (http://www.resdc.cn); Socioeconomic and policy environment data were obtained from the National Economic and Social Development Statistics Bullet in 2021 and the Government Work Report 2021 of the districts and counties of the YRD city cluster, and the data included in resource endowment are from the official website of the State Forestry and Grassland Administration (http://www.forestry.gov.cn) and the official websites of other relevant departments.

## 3 Results

### 3.1 Characteristics of the evolution of spatial distribution patterns

The ArcGIS 10.8 software was used to calculate the nearest neighbor index of high-level Class A tourist attractions in the YRD city cluster in 2001, 2006, 2011, 2016, and 2021 (Table 1). Except for 2001, the R-value of the nearest neighbor index of high-level Class A tourist attractions in the YRD city cluster for the remaining four time periods is <1, and all show a clustered spatial distribution pattern. From the temporal change of R-value viewpoint, the R-value exceeded 1 in 2001, with 0.059 P-value and 95% confidence level. During that time, there were 16 high-level Class A tourist attractions in the YRD city cluster, sporadically distributed in various cities and in a discrete distribution. In 2006, 2011, 2016, and 2021, the R-value gradually decreased in fluctuation. All passed the 99% confidence level, indicating that the spatial distribution of high-level Grade A tourist attractions in the YRD city cluster exhibited a significant clustering trend.

**Table 1:** Nearest neighbor index of high-level Grade A tourist attractions in the Yangtze River Delta City Cluster.

Year	Number of Scenic Spots	Observations of Closest Neighboring Distances (m)	Theoretical Nearest Neighbor Distance (m)	Nearest Neighbor Index (R)	Spatial Distribution Patterns
2001	16	48,206.55	38,671.16	1.2466	Dispersed
2006	124	15,663.34	23,368.49	0.6703	Clustered
2011	411	9,187.55	13,655.15	0.6728	Clustered
2016	851	6,800.99	10,024.04	0.6785	Clustered
2021	1,483	5,148.17	7,801.59	0.6599	Clustered

### 3.2 Characteristics of spatial distribution density evolution

**Fig 2:**
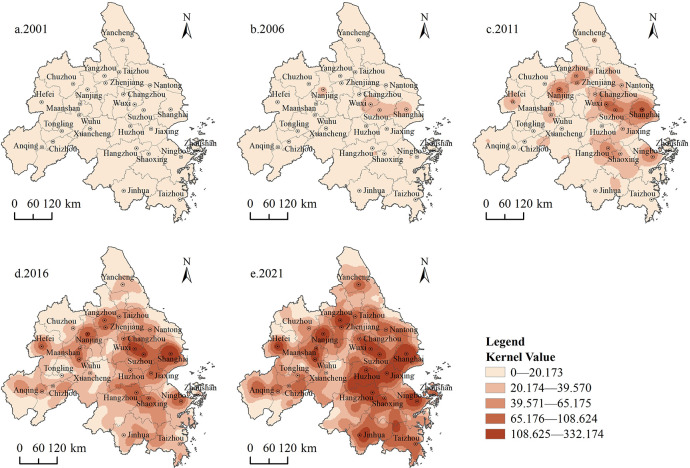
Kernel density map of high-level Grade A tourist attractions in the YRD city cluster.

**Fig 3:**
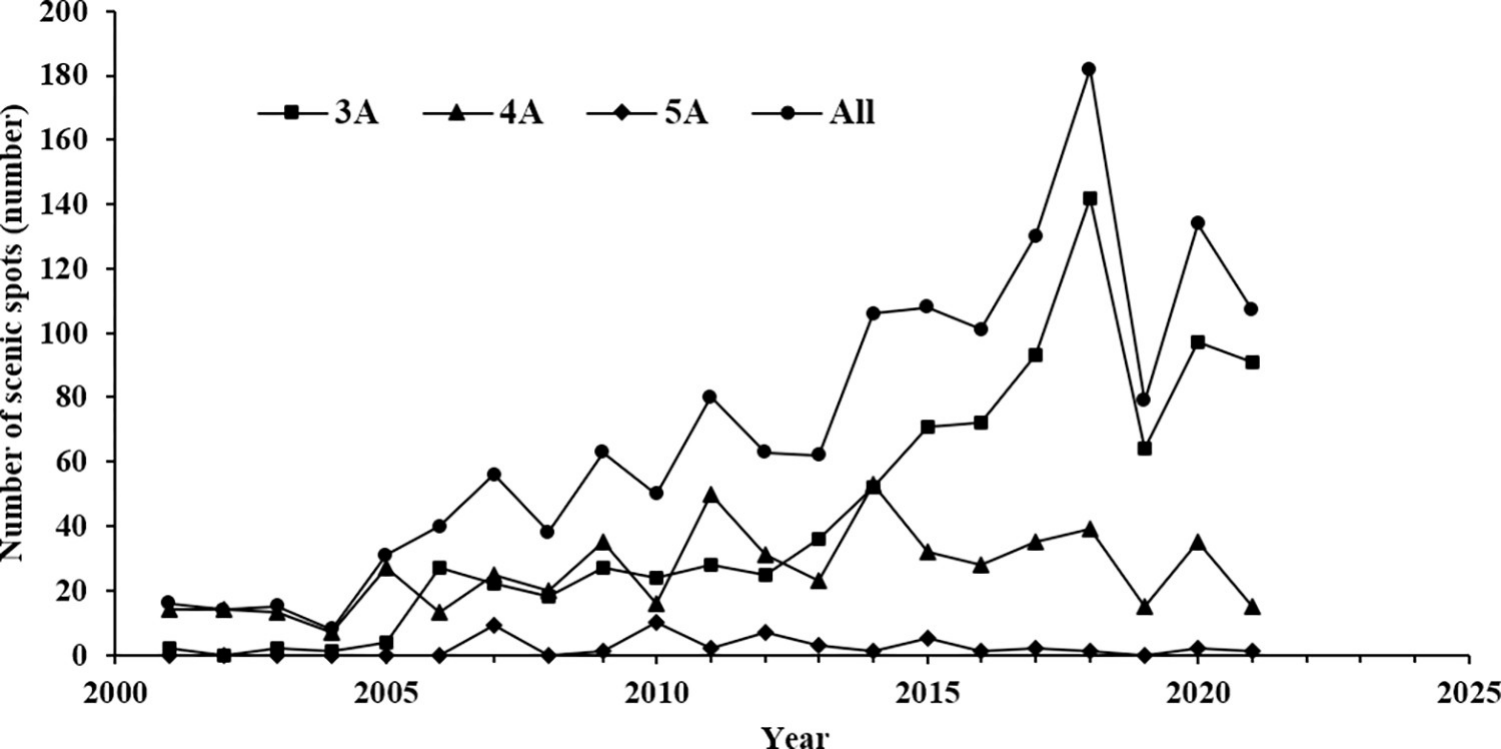
Changes in the number of high-level Grade A tourist attractions in the YRD city cluster.

Using the kernel density estimation method, the spatial distribution density of high-level Grade A tourist attractions in the YRD city cluster was visualized and analyzed over five time periods (Fig 2). The overall trend of the kernel density of the tourist attractions in the five periods is from “point-like dispersion to regimentation structure,” and a high-density distribution area in the shape of a “B-type” is gradually formed. In 2006, there were only 124 high-level Grade A tourist attractions in the YRD city cluster, a relatively small number (Fig 3). The higher kernel densities were only sporadically distributed in Nanjing, Suzhou, and Shanghai. In 2011, based on the stable growth of kernel densities in Nanjing, Suzhou, and Shanghai, high-density distribution points such as Ningbo, Hangzhou, and Hefei were newly added. In 2016, the clustering range of high-level Grade A tourist attractions was further expanded, with a significant increase in high-density areas that spread from the center of the high-density value step by step to the surrounding area, with the highest nuclear density value of 197.852 per 10,000 km^2^ and the greatest increase in the east-central part of the YRD urban agglomeration. In 2021, the original scope of the scenic area expanded significantly, with a “point-like” distribution gradually extending to the surrounding radiation to form a “group” distribution. The region appeared in Huzhou, Jiaxing, Jinhua, and Yangzhou, representing the high-density centralized contiguous area, a “B” nuclear density structure.

### 3.3 Evolutionary characteristics of spatial distribution directions

**Fig 4:**
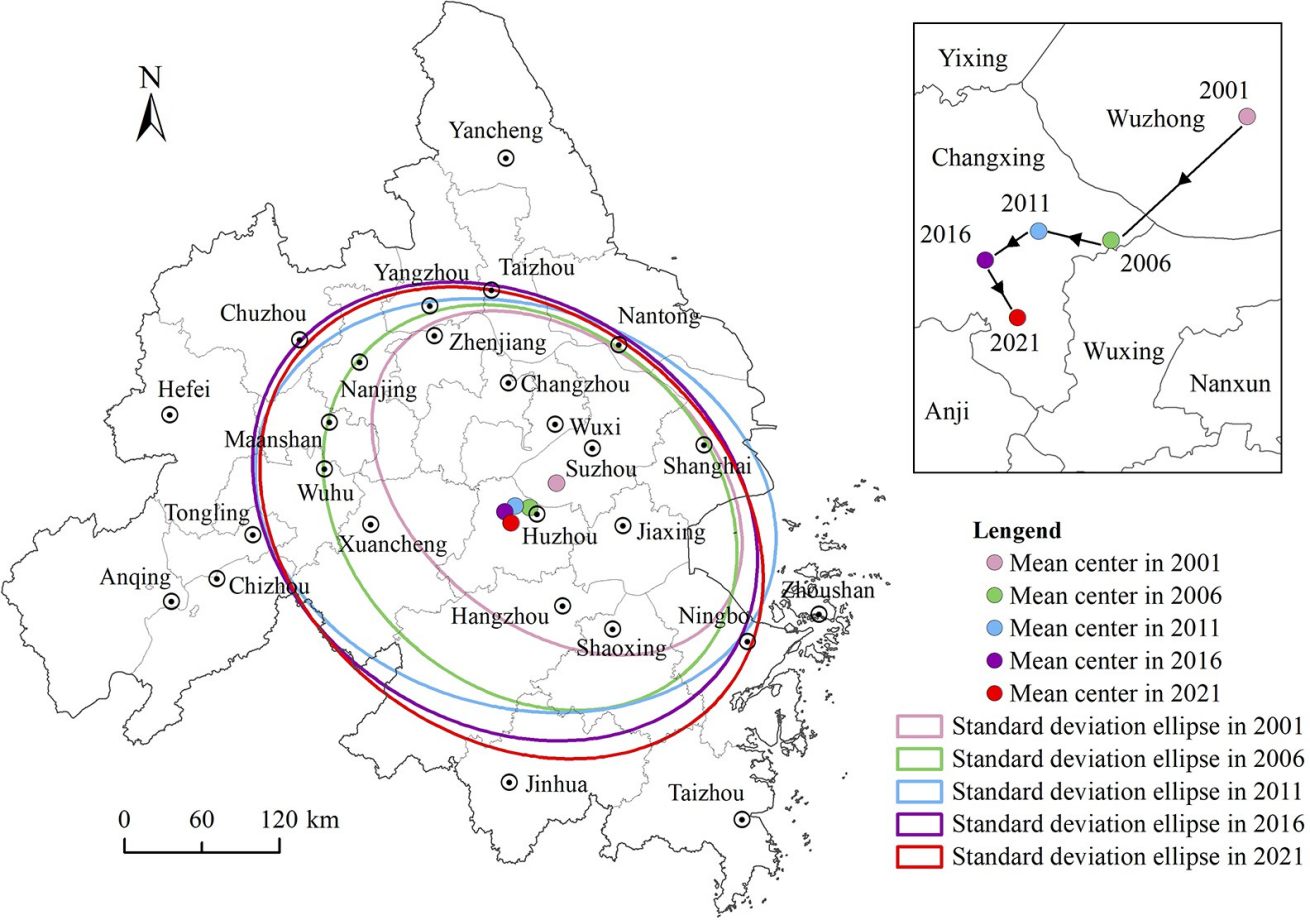
Ellipse plot of standard deviation of high-level Grade A tourist attractions in the YRD city cluster.

The standard deviation ellipse visually expresses the directionality and evolutionary trend of the spatial distribution of high-level Grade A tourist attractions in the YRD city cluster (Fig 4). From the perspective of the spatial and temporal distribution’s center of gravity, the center of scenic spot distribution in five time periods varies between 119.828°E—120.265°E and 30.854°N—31.092°N and is roughly located at the junction of Suzhou and Huzhou. From the perspective of the moving trajectory of the mean center, it is moving in a southwest direction as a whole, and has moved nearly 47.25km during the 20-year period. Additionally, the distance moved in the east–west direction was greater than in the north–south direction.

Regarding the change in the area of the standard deviation ellipse, the area showed year-on-year growth during all five time periods, indicating that the distribution of high-level Grade A tourist attractions in the YRD city cluster is gradually expanding. In 2001, the standard deviation ellipse basically covered the entire area of Jiaxing, Huzhou, Suzhou, Wuxi, Changzhou, Zhenjiang, and part of Nanjing, Shanghai, Shaoxing, and Ningbo; in 2006, the coverage had increased compared with 2001, including part of Taizhou, Maanshan, Xuancheng, and Hangzhou; and in 2011, based on keeping the original scope unchanged, part of Nantong, Yangzhou, and Wuhu were added. In 2016, the standard deviation ellipse was added to parts of Chuzhou and Jinhua compared to 2011. In 2021, the area of the standard deviation ellipse changed less significantly compared to 2016 and continued to expand the coverage of Jinhua and Shaoxing; however, the coverage of Shanghai and Suzhou was reduced. Overall, the distribution range of high-level Grade A tourist attractions in the YRD city cluster changed considerably from 2001 to 2011, yet changed less from 2011 to 2021.

From the change in the angle of turn of the standard deviation ellipse, on the whole, the distribution of high-level Grade A tourist attractions in the YRD city cluster shows a northwest–southeast spatial pattern. Generally, it turns in a counterclockwise direction, with the angle of turn θ decreasing from 129.010° to 127.311°, indicating an enhanced spatial distribution pattern. Meanwhile, the values of both the long and short axes of the standard deviation ellipse show an increasing trend during the 20-year period, reflecting a particular diffusion of high-level Class A tourist attractions in the east–west and north–south directions.

### 3.4 The evolution of spatial distributional correlations

**Fig 5:**
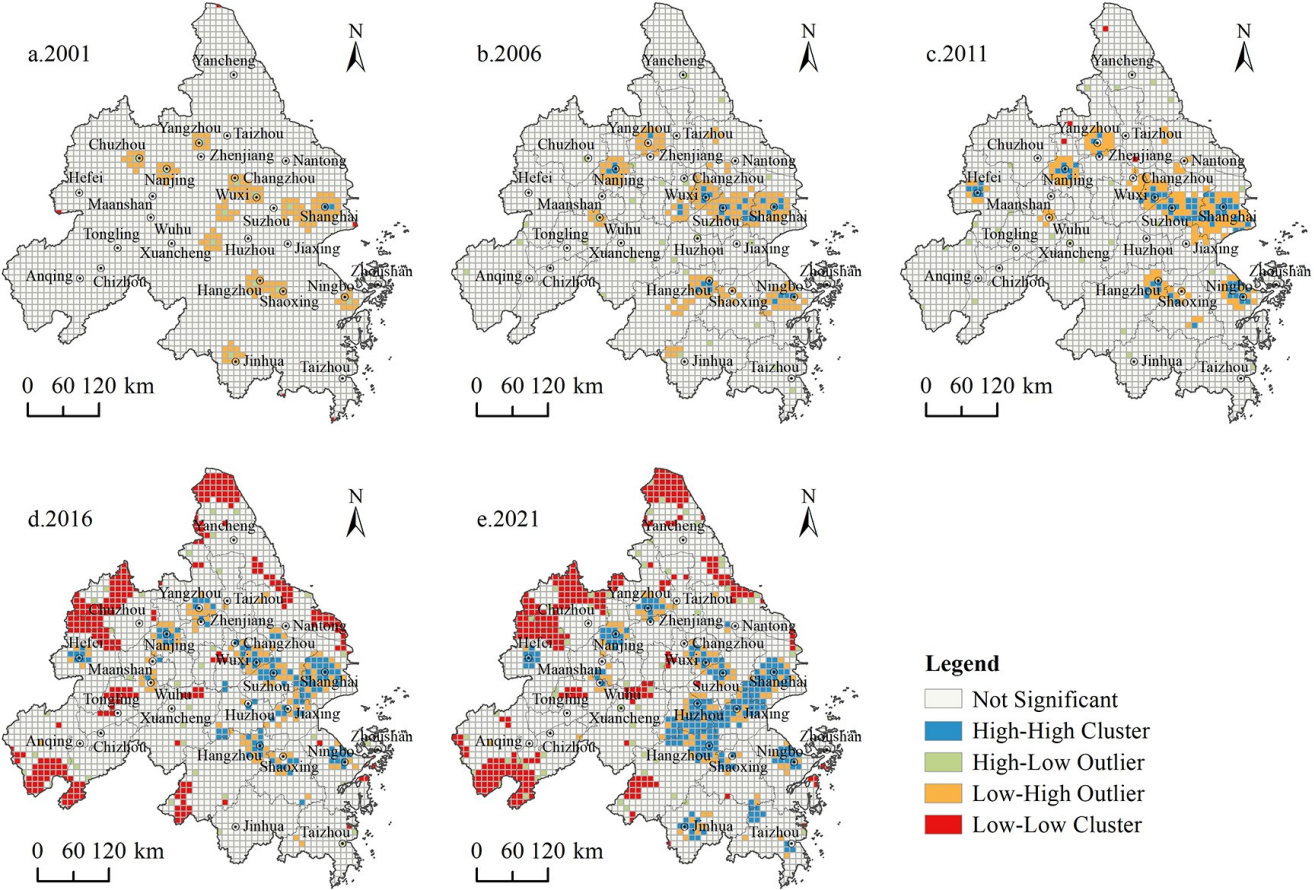
Lsia map of high-level Grade A tourist attractions in the YRD city cluster.

By repeatedly comparing the spatial correlation features at different scales, with the help of the creation of a fishing net tool in ArcGIS 10.8 software, we constructed a 10 × 10 km^2^ grid of the YRD urban agglomeration and applied the spatial autocorrelation method to analyze the spatial correlation relationship of high, A-level tourist attractions in the region. Postcalculations, the global Moran’s *I* of the five time periods are all positive, at 0.0532, 0.1220, 0.1761, 0.1728, and 0.2249, respectively. Additionally, the *P*-value passes the 99% significance level test, indicating strong positive correlations in high-level Class A tourist attractions in the YRD city cluster, indicating a strong agglomeration pattern. Furthermore, the value gradually increases, proving that the spatial clustering trend continues to gradually increase. To further identify the local correlation areas of the spatial differentiation of scenic spots in each period, LISA significance clustering maps of high-level Grade A tourist scenic spots in the YRD city cluster from 2001 to 2021 were drawn (Fig 5). Overall, the significant regions of local spatial autocorrelation of high-level Class A tourist attractions in the YRD city cluster during the study period include three types: high-high (H-H), low-low (L-L), and low-high (L-H), with a “spatial club convergence.” From the distribution of the types, H-H agglomerations are concentrated in Shanghai, Jiaxing, Suzhou, and Huzhou, etc.; L-L agglomerations are concentrated in Yancheng, Hefei, Chuzhou, Chizhou, and Anqing, etc.; and L-H agglomerations are adjacent to the distribution of H-H agglomerations, with a concentrated layout in Yangzhou, Nanjing, Changzhou, Ningbo, and other vast areas. From 2001 to 2011, L-H agglomerations dominated, while H-H and L-L agglomerations did not appear in the region for the time being. From 2011 to 2021, the number of L-H agglomeration units began to decline significantly, the number of H-H and L-L agglomeration units increased from 77 and 4 to 205 and 363, respectively, and high-value agglomerations and low-value agglomerations began to spread out to the periphery, showing a growth trend. Generally speaking, the spatial pattern of high-level Class A tourist attractions in the YRD city cluster is based on “homogeneous associations, supplemented by heterogeneous associations,” with high-value associations concentrated in the east-central part of the region and low-value associations in the periphery of the region. The spatial evolution paths followed “incremental transfers, supplemented by cross-level transfers.”

## 4 Analysis of the spatiotemporal evolutionary driving mechanism of high-level Class A tourist attractions

### 4.1 Identification of influencing factors

The spatial distribution and temporal change of high A-level tourist attractions are due to several factors. The natural environment is the basic condition for forming scenic spots, and an excellent natural environment is favorable for forming scenic spots and vice versa [41]. Resource endowment is the primary guarantee of scenic spot development, the so-called “no mountain, no water, difficult to construct the scene.” For developing the scenic spots, the first choice is an area rich in tourism resources [46]. A social economy is an important driver of scenic evolution; the larger the population size of the tourist destination, the better the economic development, transportation, industrial structure, and development of the local tourism industry. The policy background is a good support for managing scenic spots. The government plays an important role in regulating and supervising the spatial distribution of scenic spots [41]. Based on the scientific nature of indicators and data availability, combined with existing studies [20,21,41,46], this study analyzes the spatiotemporal evolutionary driving mechanism of high-level Grade A tourist attractions in the YRD urban agglomeration from four aspects: natural environment, resource endowment, socioeconomics, and policy context (Table 2).

**Table 2:** Impact factors and indicator determinants.

Target Level	Standardized Layer	Indicator Layer	*%IncMSE*
Natural Environment	Terrain elevation	DEM mean (*X*_1_)	19.8070
	Water conditions	River network density (*X*_2_)	12.4564
	Atmospheric conditions	Average annual temperature (*X*_3_)	10.5867
Resource Endowment	Natural and humanistic tourism resources	Number of national scenic spots, national forest park, national geopark, national key cultural relics protection units, national intangible cultural heritages, historical and cultural cities, towns and villages, traditional villages, and villages with minority characteristics (*X*_4_)	28.9555
Socioeconomic	Transport conditions	Road density (*X*_5_)	11.8970
	Economic level	GDP (*X*_6_)	8.7704
	Industrial structure	Value added of tertiary sector as a share of GDP (*X*_7_)	3.5237
	Customer potential	Resident population (*X*_8_)	8.6140
Policy Context	Industrial support	Number of mentions of “tourism” in government work reports (*X*_9_)	5.3332

Note: *%IncMSE* is the increase in mean squared error; a larger value indicates a more significant variable.

### 4.2 Analysis of influencing factors

**Fig 6:**
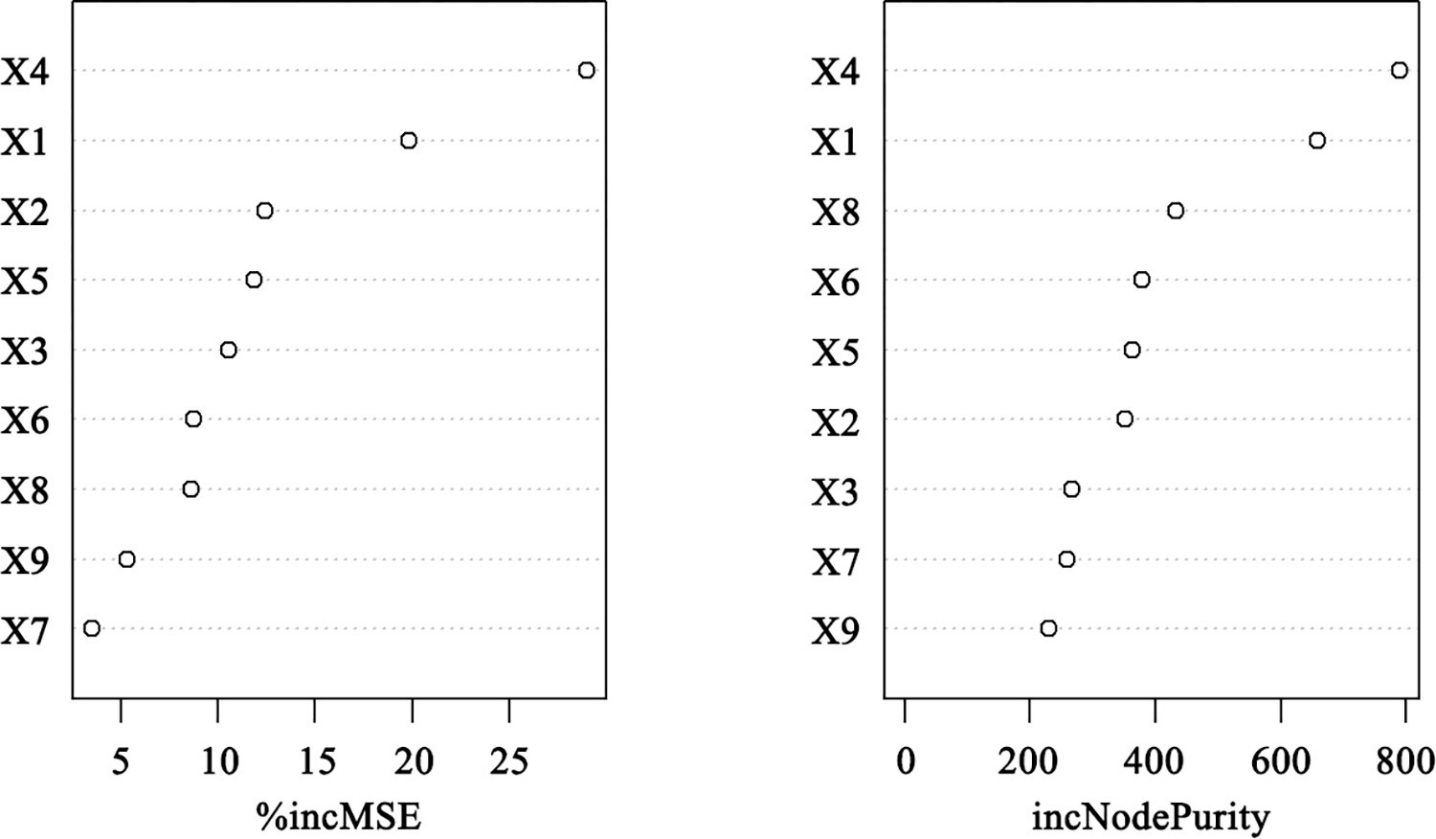
Importance ranking chart of influencing factors.

Results of the random forest model showed that *R*^2^ = 0.89, MAE = 1.43, indicating a high degree of model fit for the analysis of influencing factors. The ranking graph of the importance of the influencing factors of the two indicators of *%IncMSE* and *IncNodePurity* is calculated by the *varImplot* function package (Fig 6), and it can be seen from the graph that the importance rankings of the two measures are not very different. Taking the result of *%IncMSE* as an example, the factors with substantial influence are, in order, the resource endowment (*X4*), DEM mean (*X1*), river network density (*X2*), road density (*X5*), average annual temperature (*X3*), GDP (*X6*), resident population (*X8*), policy context (*X9*), and the value added of tertiary sector as a share of GDP (*X7)*.

**Fig 7:**
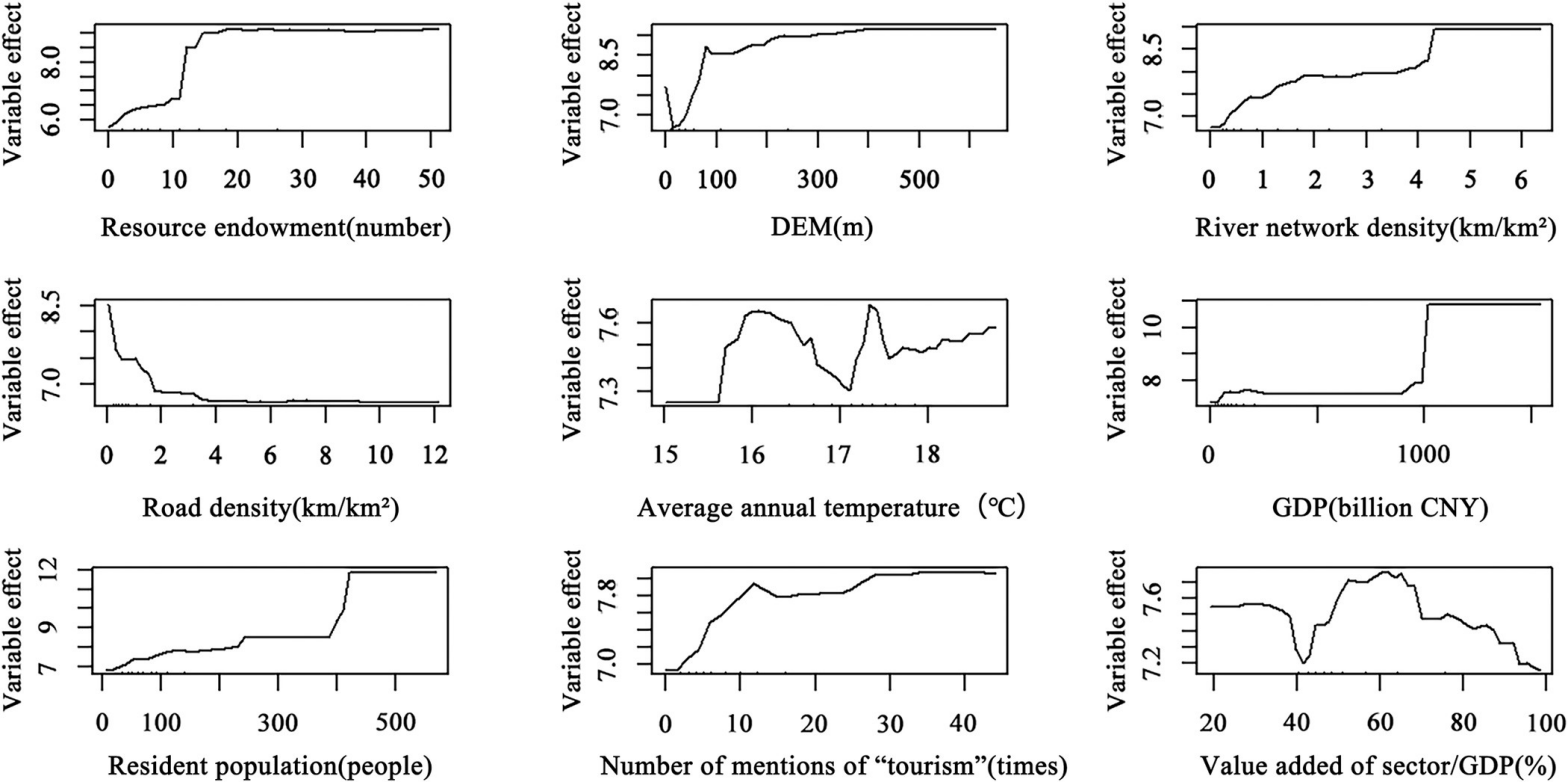
Map of how influencing factors contribute to the distribution of high-level Class A tourist attractions.

Furthermore, the *partialPlot* function package was used to calculate the partial dependence of each influencing factor and the number of scenic spots for revealing the nonlinear relationship between each factor and the distribution of scenic spots (Fig 7) and influencing each factor on the distribution of scenic spots is complex and nonlinear.

The top six influencing factors are selected for specific analysis; except for the negative relationship between road network density and the number of tourist attractions, the remaining indicators are positively related to the number of tourist attractions. The influence of resource endowment on the distribution of scenic spots is the first. The degree of positive correlation between resource endowment and the number of tourist attractions presents differentiated characteristics under different thresholds. When the resource endowment is less than 20, it plays a strong role in the distribution of tourist attractions, but when it exceeds 20, the number of tourist attractions stays at a stable level, and the influence of resource endowment on the distribution of tourist attractions eases slightly. Tourism resources are the core foundation of tourist attraction development; the more tourism resources in the area, the greater the chance of tourist attraction development and the number of tourist attractions. The influence of the DEM and the effect of the river network density on the distribution of tourist attractions are second and third, respectively. Specifically, the number of tourist attractions grows faster when the DEM is less than 100 meters, the average elevation of the YRD area is generally low, a large number of tourist attractions are distributed in the low elevation zone, and only a few natural tourist attractions are scattered at higher elevations. The river network density always maintains a stable positive correlation with the number of tourist attractions, and the rich water conditions create a favorable environment for the formation of tourist attractions. The influence of the road network density on the distribution of tourist attractions is ranked fourth, and its relationship with the distribution of tourist attractions is negative. With the increase in road network density, the number of tourist attractions is decreasing, especially when the density of the road network is less than 4 km/km^2^. The negative correlation between the two is significant. Of all the high-level Grade A tourist attractions in the YRD city cluster, more than 55% belong to the categories of history, culture, nature, and ecology. Due to cultural and ecological environmental protection considerations, the construction of roads leading to tourist attractions has been restricted in some areas to further protect the integrity of cultural heritage and ecosystems. The influence of average annual temperature and the effect of GDP on the distribution of tourist attractions are fifth and sixth, respectively. As the temperature rises, the number of tourist attractions gradually rises overall despite fluctuations, and the average annual temperature of the YRD region as a whole is maintained at around 17°C, so a slight increase in temperature does not affect the construction of tourist attractions; on the contrary, it promotes the development of tourist attractions, thus increasing the number of tourist attractions. When the GDP exceeds 1000 billion CNY, the number of tourist attractions shows a trend of rapid growth. The higher the GDP, the more capable the region is of constructing tourist attractions. The YRD city cluster is one of the foremost regions in China in terms of economic strength, and the region’s advanced infrastructure, strong demand for tourism, and high level of consumption power provide strong support for the development of tourist attractions.

### 4.3 Driving mechanism analysis

**Fig 8:**
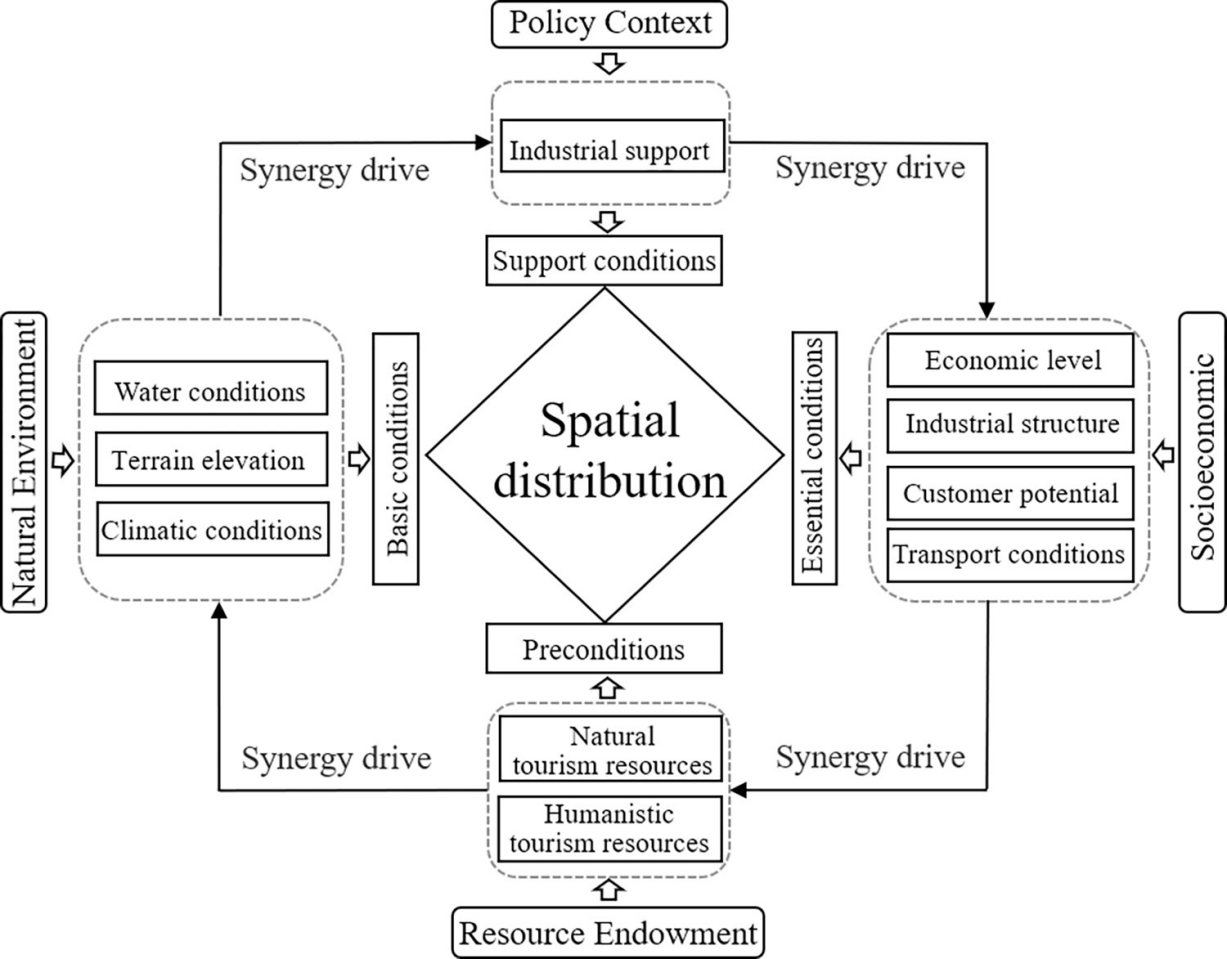
Mechanisms influencing the spatial pattern of high-level Grade A tourist attractions in the YRD city cluster.

The spatial distribution and change of high A-level tourist attractions result from the comprehensive role of natural and humanistic elements. The spatial distribution of scenic spots results from the dynamic game between different elements, and the correlations between scenic areas and other elements, as well as the same element and different types of scenic areas, are heterogeneous [45]. Topography and geomorphology have largely constraining effects on the distribution of scenic spots. Additionally, altitude vaguely determines the type of scenic spots. Natural resource scenic spots are mostly distributed in mountainous and hilly areas, while human resource scenic spots are widely distributed in the plains [43]. As a natural tourism resource type, river systems are a strong gravitational element, influencing the development of human civilization, and an important condition for creating and developing high-level scenic spots in the region. YRD areas have the highest density of river networks in China, and the water conditions of the urban agglomeration of the YRD are richer than the other regions. In this special natural geographic environment, the river system affects the quality of scenic tourism resources and guarantees the cultivation of a large-scale tourist market in the region. Different forms of water bodies play their shape, shadow, sound, color, sweetness, strangeness, and other aesthetic functions to form a rich and colorful water landscape, attracting tourists [45]. Moreover, many tourists are attracted by the warm and comfortable climate that stimulates human production and life and come to enjoy the experience. Furthermore, the appropriate temperature cultivates several high-quality natural landscapes, affecting the development high-level Class A tourist attractions. Tourism resource endowment is the foundation of scenic spot development, and the number of tourist attractions in areas with greater resources tends to be higher than in other places. The YRD city cluster has a long history and culture in the middle and lower reaches of the Yangtze River. Jinling, Wu-Yue, Huaiyang, and Hui cultures have exchanged, fused, and precipitated, forming several high-quality human tourism resources. In addition, there are >1,500 national intangible cultural heritage sites, minority villages, and key cultural relic protection units. High-quality natural resources and rich cultural heritage have created a favorable environment for developing high-level Grade A scenic spots in the region, important for the coordinated tourism development in the YRD city cluster. With mass tourism and the shift to recovery from the epidemic, socioeconomic conditions have strongly driven regional tourism. The size of the population in the region is an important source of tourist attractions; transportation infrastructure brings tourist destinations closer to their sources and enhances the accessibility of tourist attractions. Tourism is an important part of the tertiary industry, and the ratio of the tertiary industry largely reflects the state of tourism development in the region. Regions with high GDPs tend to have more developed infrastructure and better service levels, affecting the distribution of tourist attractions and scenic spots (Fig 8).

## 5 Conclusions and suggestions

### 5.1 Conclusions

Using the GIS spatial analysis, this study analyzes the evolutionary characteristics of the spatial pattern of high-level Grade A tourist attractions in the YRD urban agglomeration, from 2001 to 2021. We explore the driving mechanisms using the random forest model. The primary research conclusions are as follows:

1. From the perspective of distribution pattern, the overall distribution of high-level Grade A tourist attractions in the YRD city cluster from 2001 to 2021 is agglomeration, and the spatial agglomeration becomes more and more significant with the evolution of time.
2. From the perspective of distribution density, from 2001 to 2021, the core density of high-level Grade A tourist attractions in the YRD city cluster evolved from “point-like dispersion to regiment-like combination” and eventually formed “Maanshan—Nanjing—Zhenjiang—Shanghai—Jiaxing—Shaoxing—Taizhou” as the connecting line of the “B” high-density structure.
3. From the direction of distribution, the overall distribution of high-level Grade A tourist attractions in the YRD city cluster from 2001 to 2021 is in the direction of “northwest–southeast,” with counterclockwise rotation, and the center of gravity of the distribution migrates to the southwest, and the location of the center of gravity migrates from Suzhou City to Huzhou City.
4. From the viewpoint of distribution correlation, there is a significant positive spatial correlation in the distribution of high-level Grade A tourist attractions in the YRD city cluster from 2001 to 2021, and the areas with significant localized spatial autocorrelation include three types: high-high (H-H), low-low (L-L), and low-high (L-H), and there is the phenomenon of “spatial club convergence.”
5. The main factors affecting the spatial and temporal distribution pattern of high-level Class A tourist attractions in the YRD city cluster include the number of highway miles at the end of the year, the proportion of tertiary industry in GDP, average annual temperature, river network density, resident population, GDP, and resource endowment. The influence of each factor on the distribution of high-level Class A tourist attractions is nonlinear and complex.

The article explores the spatiotemporal evolution and driving mechanisms of high-level Class A tourist attractions in the YRD city cluster; however, the selection of influencing factors still needs improvement. There is a notable difference between natural and humanistic tourist attractions concerning the distribution law and influencing factors; however, they are not distinguished in this study. At the same time, due to data availability and other reasons, this paper only selected one year’s cross-section statistics to analyze the influencing factors, failing to explore the time change of the influencing factors from a more precise perspective; this needs to be improved in this paper. Therefore, in the context of high-quality development in tourism, distinguishing the evolution law of natural and humanistic scenic spots, constructing a more targeted index system of influencing factors, and revealing the changes in its time dimension are the directions that need to be explored in depth in the next step.

### 5.2 Suggestions

This study provides a better portrayal of the spatial and temporal evolution of high-level Grade A tourist attractions in the YRD city cluster from the spatial measurement perspective. Results show there are still many issues in developing high-level Grade A tourist attractions in the YRD city cluster. As uncertainty about global tourism has increased due to the impact of COVID-19, coupled with the transformation of the contradictions of domestic society, the imbalance and insufficiency of tourism development are prominent. Thus, measures must be taken to continuously improve the high-quality development of the tourism industry in the YRD city cluster.

1. Improve the layout and integrate development: Under the influence of the YRD integrated development strategy and culture and tourism, creating a new path for integrated tourism development through resource sharing, integration of differences, and brand cocreation. The YRD city cluster should integrate differentiated tourism resources, focusing on creating a Shanghai “urban tourism” model benchmark, the high-level construction of a cultural tourism demonstration area in southern Anhui, and an Anhui leisure tourism pioneer area. Efforts to promote the construction of an ecological and cultural tourism area along the Taihu Lake in southern Jiangsu Province and northern Zhejiang should give complete play to their own resource advantages and vigorously develop ecotourism and waterfront tourism. Thus, we should accelerate the constructing tourism and transportation services for the YRD city cluster, launching a special tourism and transportation line for the YRD city cluster, planning cross-regional tourism boutique lines, and building a demonstration base for high-quality tourism in the YRD city cluster. Additionally, we should strengthen the cooperation of tourism market entities and further improve cultural and tourism initiatives for the benefit and convenience of the people in the YRD city cluster.
2. Optimize supply and quality services: To promote mass tourism, the structural reform of the supply side of tourism should be implemented in a coordinated manner, the stock of tourism resources should be revitalized, the supply of high-quality incremental supply should be improved, and the efficient, high-quality, balanced tourism development public services should be strengthened. Additionally, we should integrate regional tourism resources, make efforts to create features and improve quality, cultivate high-quality tourism products, build diversified tourism forms, give full play to the driving function of the core node in Shanghai, jointly build a transportation and distribution hub with Hefei, Nanjing, and Hangzhou as the main nodes, and improve the tourism transportation network. The following key construction projects for integrated tourism development in the YRD city cluster should be implemented to drive the supply of tourism products in the YRD city cluster to achieve quality upgrading: open up cooperation, look at Huangshan, promote the construction of the Hangzhou-Huang world-class natural ecological and cultural tourism corridor, and create an internationalized tourism demonstration circle of ancient towns and villages in the southern water towns of Jiangnan In major node cities, we should build cultural and tourism service centers, tourist information centers, and other public service windows to improve tourist travel satisfaction.
3. Digital empowerment and modern governance: Thanks to the good infrastructure and comprehensive strength of the region, developing “smart tourism” in the new era is possible. We will create new, comprehensive “Internet+” digital tourism scenarios, introduce digital services such as electronic maps, voice guides, route recommendations, and cloud reservations, cultivate new forms of tourism, such as live cloud tours and online excursions, and support the innovation of scientific and technological tourism products to create integral experience projects, such as live interpretation and holographic projections. Big data should be taken as the core and the government, enterprises, society, and other parties should be coordinated to build a modernized, technological, and intelligent tourism governance system, explore the digital integrated supervision system, and realize precise supervision in the field of tourism.

## Supporting information

S1 File List of high-level Grade A tourist attractions in the Yangtze River Delta City Cluster. (XLS)
